# Evaluation of Hydrophobic Nanoparticulate Delivery System for Insulin

**DOI:** 10.4103/0250-474X.49091

**Published:** 2008

**Authors:** P. S. Singnurkar, S. K. Gidwani

**Affiliations:** USV Limited, B. S. D. Marg, Govandi, Mumbai-400 088, India

**Keywords:** Insulin, stearic acid, nanoparticles, solvent diffusion, oral delivery

## Abstract

Insulin loaded hydrophobic nanoparticles were prepared by solvent diffusion followed by lyophilization. Nanoparticles were characterized for mean size by dynamic laser scattering and for shape by scanning electron microscopy. Insulin encapsulation efficiency, *in vitro* stability of nanoparticles in presence of proteolytic enzymes and *in vitro* release were determined by high pressure liquid chromatography analysis. The biological activity insulin from the nanopraticles was estimated by enzyme-linked immunosorbant assay and *in vivo* using Wister diabetic rats. Nanoparticles ranged 0.526±0.071 μm in diameter. Insulin encapsulation efficiency was 95.7±1.2%. Insulin hydrophobic nanoparticles suppressed insulin release promoted sustained release in pH 7.4 phosphate buffer and shown to protect insulin from enzymatic degradation *in vitro* in presence of chymotripsin. Nanoencapsulated insulin was bioactive, demonstrated through both in vivo and *in vitro*.

Oral delivery formulation for insulin is highly desirable from a patient compliance point of view; however only a small portion of insulin administered orally reaches the blood stream, mainly due to extensive degradation of the protein in the gastrointestinal tract. The various challenges associated with the oral delivery of proteins usually are evaluated by determining the fate of the protein in the gastrointestinal tract (GIT). The main challenges reported are enzymatic degradation and a lack of sufficient insulin permeability through the GIT. The enzymatic barrier and epithelial barrier for proteins have been reviewed in detail elsewhere^1^,^2^. Further, large size and hydrophilicity of the molecule greatly limits its transport across the intestinal epithelium^3^. No specific transport mechanism is present for the passage of insulin to cross the intestinal cell monolayer. Insulin molecules that are unable to cross the intestinal barrier thus get further exposed to the intestinal proteolytic activity; this in turn reduces the bioavailability of the protein. In spite of the obstacles to oral delivery, substantial evidence suggests that pharmaceutical polypeptides are absorbed through the intestinal mucosa, although in minute amounts^4^. Fatty acids have been shown to enhance the permeability of peptide drugs. The mechanism whereby the permeability of peptide drugs was enhanced by the fatty acid association with the disorder in the membrane's interior of these fatty acids with polar head group of phospholipids^5^–^7^.

The aim of this study was to prepare and characterize insulin loaded hydrophobic nanoparticles with combined use of stearic acid, a tocopherol acetate and soya phosphatidyl choline (SPC) in presence of zinc and hydroxypropyl-β-cyclodextrin (HP-β-CD) to evaluate improvement in oral bioavailabilty of insulin. A drug carrier for insulin should provide a stable and biocompatible environment to ensure that the main fraction of the therapeutic protein will be biologically active following encapsulation. In this work we have demonstrated oral absorption of insulin in the form of hydrophobic nanoparticles as a part of complex formulation system to collectively improve the stability and oral bioavailability of insulin when administered to streptozocin induced diabetic rats. The process for the preparation of nanoparticles greatly influence the encapsulation efficiency and stability of the protein drug, especially when protein molecule comes in contact with hydrophobic surfaces, air-water interfaces, shear stress, temperature and organic solvents^8^,^9^. In addition, size of the nanoparticles has been shown to have influence on the oral absorption^10^,^11^. In this work, process was optimized to produce well controlled particle size and high encapsulation efficiency of insulin in the hydrophobic nanoparticles. Lyophilization technique for the preparation of Insulin nanopraticles was designed to improve to the stability and encapsulation efficiency of insulin in the hydrophobic nanoparticles. Nanoparticles were characterized in terms of size distribution, morphology, encapsulation efficiency, *in vitro* insulin release behavior and *in vitro* and *in vivo* biological activity of insulin following encapsulation.

## MATERIALS AND METHODS

Human insulin of recombinant DNA origin was obtained from Akzo Nobel, Mumbai. m-Cresol, hydrochloric acid 36% (HCl), anhydrous zinc chloride, Tris buffer and t-butanol were all procured from Merck India, Mumbai. Hydroxypropyl-β-cyclodextrin (HP-P-CD) was from Cerestar®, sodium hydroxide pellets were from S. D. Fine Chemicals, Boisar, stearic acid was from Cognis, soya phosphatidylcholine (SPC) was from Lipoid AG, α tocopherol acetate was from Sigma Aldrich, St. Louis, MO, USA, α-chymotripsin was from HiMedia, Mumbai and human insulin ELISA kits were from Mercodia®.

### Preparation of hydrophobic nanopraticles of Insulin:

Human Insulin solution (50 IU/ml) was prepared in tris buffer pH 7.4 (10 mmol) containing 0.8 mg/ml m-cresol and mixed with solution of HP-β-CD (24 mg/ml) in tris buffer pH 7.4 (10 mmol) in a beaker and stirred for 10 to 15 min on magnetic stirrer. Then zinc chloride solution (0.4 mg/ml) was added in the form of micro-droplets to the above insulin HP-β-CD solution with continuous stirring, after complete addition solution was stirred for further 10 to 15 min to obtain opalescent solution of insulin. The above insulin-zinc mixture was mixed with solution of stearic acid (0.5% w/v), α tocopherol acetate (0.04% w/v) and SPC (0.04% w/v) in t-butanol:water mixture (9:1) solution in a beaker with stirring for 5 min. The resulting opalescent solution was frozen at −40° for 3 h in a tubular glass vial and freeze dried using table top laboratory freeze dryer (Lanconco®, USA) for 36 h. After complete drying, vacuum was released with nitrogen gas to allow purging of vials with nitrogen which were then immediately capped with butyl rubber stoppers and aluminum seal. Sealed vials were stored at 2-8°.

### RP-HPLC analysis of insulin:

Insulin analysis was performed by gradient controlled HPLC equipped with UV/Vis detector (Agilent 1200 series RRLC®) and a reversed phase C_18_, 1.8 μm, 4.6×50 mm column. The gradient system consisted of mobile phase-A, mixture of buffer and acetonitrile (82:18), and mobile phase-B, mixture of buffer and acetonitrile (50:50). The flow rate was 1 ml/min with oven temperature of 40° and detection wavelength at 214 nm. The nanoparticles obtained after lyophilization were dissolved in a mixture of 0.01N HCl and ethanol (99%) with solvent ratio of 4:1, respectively. The solution was filtered though 0.22 μm syringe disc filter (Ultipor®N66, Pall life Sciences) and injected.

### Characterization of nanoparticles:

Samples of nanoparticles were diluted with 0.22 μm filtered tris buffer pH 5.5 solution, then by dynamic laser light scattering (Brookhaven Instruments BI 90 Particle sizer, Holtsvilee, New York) at room temperature. The laser power is 5 mW HeNe vertically polarised. The morphology of the human insulin nanoparticles was viewed using a conventional scanning electron microscope (JSM 5400, Joel, Japan) at an accelerating voltage of I5 kV. One drop of the nanoparticle suspension was placed on a graphite surface. After air drying, the sample was coated with gold using Ion Sputter.

### Insulin entrapment efficiency:

The entrapment efficiency was measured by dispersing human insulin nanoparticles in 0.01N HCI and mixed for 5 min on vortex mixer (Remi®, India). The mixture was ultracentrifuged in order to isolate entrapped insulin from unentraped insulin. The supernatant was removed and nanoparticle sediment was washed by distilled water. Sediment was dissolved in a mixture of 0.01N HCl and ethanol (99%) with solvent ratio of 4:1, respectively. The solution was sonicated and filtered through 0.22 μm syringe disc filter (Ultipor®N66, Pall life Sciences). The filtered solution was quantitatively analyzed for human insulin by RP-HPLC. The entrapment efficiency (%) was determined by human insulin content obtained as percentage of initial amount used in the formulation.

### *In vitro* insulin release study:

The release rate of human insulin from the nanoparticles were determined by suspending weighed amount of lyophilized nanoparticles (equal to 100 IU of human insulin) in 50 ml, pH 7.4 phosphate buffer USP. During the experiment (3 h) samples were shaken horizontally in a constant temperature shaker (Remi®, India) at 37±1° and 50 stokes per minute. At scheduled time intervals, 1 ml of sample was removed and replaced with 1 ml of fresh release medium. The sample was ultracentrifuged and supernatant was collected and quantitatively analyzed for human insulin content by RP-HPLC.

### *In vitro* stability of insulin nanoparticles in presence of proteolytic enzymes:

The protection of insulin nanoparticles was evaluated in α-chymotrypsin with specific activity of 51 U/mg protein at pH 7.8 (optimal pH for enzyme activity) and 37±1°. The nanoparticles were suspended in 0.05 M phosphate buffer pH 7.8 to which α-chymotrypsin had been added. α-Chymotrypsin was also added to insulin solution (as control). The final concentration of the insulin and the enzyme were 15 U/ml and 26 U/ml. Samples were removed after 30 min and 0.5 ml 0.1% TFA was added to inactivate traces of enzyme present. Subsequently, 0.5 ml of ethanol 99% was added to each tube and sonicated for 10 to 15 sec. Then the volume was adjusted to get the human insulin concentration of 0.5 to 0.6 IU/ml with a mixture of 0.01N HCl and ethanol (4:1). The insulin solution was filtered though 0.22 μm syringe disc filter. The filtered solution was quantitatively analyzed for human insulin content by RP-HPLC.

### Biological activity evaluation of entrapped human insulin:

Biological activity of entrapped human insulin in the nanoparticles was assessed using ELISA technique. Insulin loaded nanoparticles (equal to 3 units of human insulin) were dissolved in a mixture of phosphate buffer pH 7.4 and ethanol 99% with solvent ratio of 4:1. The solution was mixed for 5 min and then sonicated for 15 sec and volume adjusted to 5 ml and filtered through 0.22 μm syringe disc filter. Second dilution was made by diluting 50 μl of filtrate to 200 ml with phosphate buffer pH 7.4 to final concentration of about 150 mU/l. An aliquot of sample was withdrawn from the solution and insulin content was analyzed by ELISA as per standard protocol. Results were obtained by reading the optical density at 450 nm using plate reader (BioRad®, model 680 microplate reader) and results were calculated using Microplate Analyst 3.0.2 software.

### *In vivo* studies:

Male Wistar rats (200-280 g) were housed in a light and temperature controlled environment. All the animal procedures were reviewed and approved by the ethical committee for animal studies. Diabetic state was induced with single injection of 40 mg/kg streptozocin (STZ) in citrate buffer (pH 4.5) via intraperitoneal route. The plasma samples were analyzed for glucose content by glucose oxygenase method (Reagent: Pinnacle Biotechnology Ltd, Mumbai) and measuring the optical density at 505 nm (Start 21 Autoanalyser). Rats were considered diabetic and included in the study when plasma glucose levels were near and above 300 mg/dl. Diabetic rats were divided into three groups, each containing 6 rats. Group-1, placebo nanoparticles (equal to 20 IU/kg of insulin) dispersed in 0.5 ml purified water was adiministered intragastrically to each diabetic rat as a control; Group-2: human insulin injection (Huminsulin-R® 40 IU/ml injection, Elly Lilly) was administered subcutaneously to each diabetic rat at a dose level of 2 IU/kg. This group served as reference standard; Group-3: human insulin nanopartic1es dispersed in 0.5 ml purified water (equal to 20 IU/kg of human insulin) was administered intragastrically to each diabetic rat. After administration of the dosage forms, 0.5 ml of purified water was administered. Prior to and at specified time intervals over 12 h period blood samples were collected from retroorbital vein in capillary tubes for serum human Insulin estimation and fluoride oxalate impregnated capillary tubes for glucose estimation. The serum samples were analyzed for the insulin content by using ELISA technique (Mercodia® Insulin ELISA kit). Optical density at 450 nm was measured within 30 min on microplate reader (BioRad®, Model 680) and human insulin concentration was calculated using Microplate Analysis 3.0.2 software. The plasma samples were mixed with glucose oxygenase reagent and the samples were incubated at 37° for 10 min for colour development. Optical density of the samples was measured at 505 nm. The glucose concentration was auto-calculated using Star 21 glucose autoanalyzer. Human insulin pharmacokinetic parameters such as C_max_, T_max_, T_½_ and AUC_0-t_ were estimated from the serum human insulin concentration versus time profile. Semilogarithmic plot of the serum human insulin concentration versus time was constructed for estimation of pharmacokinetic parameters such as elimination rate constant, K_e_ and plasma half life, T_½_.

Blood glucose concentrations were determined in triplicate prior to dosing and the mean concentration was considered as 100% level. All following concentration-time data were expressed as a fraction of the base line, considering the fact that blood glucose concentrations over 12 h following intragastric administration of placebo nanopartic1es to diabetic rats (control groups) were not significantly different from the base line (assuming a flat base line). The mean±SD of each concentration-time point in each treatment group was calculated and compared.

The mean of percent blood glucose concentrations at each time point was subtracted from 100% and the area above the percent blood glucose-time curve (AAC_0-12h_) were estimated by the trapezoidal rule reported by Touitou and Rubinstein^12^.

## RESULTS AND DISCUSSION

**Fig. 1:**
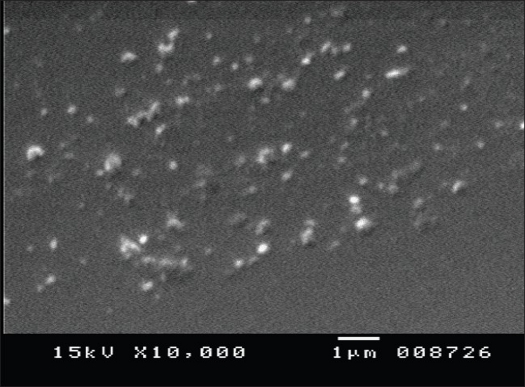
Scanning electron microscopy of insulin nanoparticles. Scanning electron micrograph of human insulin nanoparticles at 10,000X, white spherical particles are observed.

After lyophilization, a white, extremely porous, fragile and light weight cake was observed. The extremely porous and fluppy cake formation after lyophilization can be attributed to the use of t-butanol as a solvent. t-Butanol is a versatile lyophilization medium as it has very high vapor pressure (26.8 mm Hg at 20°) and high freezing point (24°)^13^. The mean diameter of the human insulin nanoparticles did not display large variation, the mean particle size was about 0.526±0.071 μm. The SEM images confirm that the nanoparticles are circular in shape and well dispersed and separated on the surface (fig. 1).

The entrapment efficiency of the nanoparticles was found to be satisfactorily high with an entrapment of 95.7±1.2%. The satisfactorily high entrapment efficiency could be attributed to the process of making the nanoparticles. It involved nucleation of insulin molecules by addition of zinc chloride solution in a controlled manner followed by entrapment into lipophilic coat using water miscible organic solvent, t-butanol followed by freezing and lyophilzation to get the nanoparticles.

**Fig. 2:**
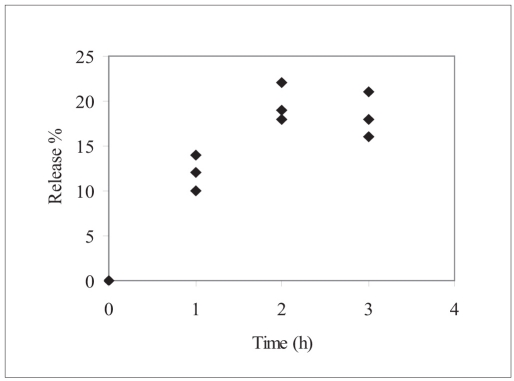
*In vitro* release behavior of insulin hydrophobic nanoparticles. ♦ Percent cumulative human insulin release with time from insulin nanoparticles in pH 7.4 phosphate buffer (n=3)

Human insulin release profile from the nanoparticles was shown in fig. 2. Each is generally characterized by slow release rate, no bust effect was observed. The possible explanation for the slow release rate in the pH 7.4 phosphate buffer USP could be due to deposition of hydrophobic coat over insulin-zinc-HP-β-CD. Degradation of insulin observed over the extended time intervals indicating the instability of human insulin to the experimental conditions.

The stability of human insulin solution and human insulin nanoparticles in presence of α-chymotripsin is depicted in Table 1. It can be seen that when insulin solution were incubated with a-chymotripsin, ≤20% of human insulin remained undegraded after 30 min. Human insulin nanoparticles incubated in the same medium, ≥80% of entrapped insulin remained undegraded over the same period. Each sample was analyzed in triplicate.

**TABLE 1:** *IN VITRO* STABILITY OF INSULIN NANOPARTICLES IN PRESENCE OF α-CHYMOTRIPSIN

Formulation	% of undegraded human insulin
Human insulin crystals	18±3
Insulin nanoparticles	83±2

Results are the mean of three observations ± SD.

Biological activity of entrapped human insulin in the nanoparticles was estimated using ELISA technique. It can be seen that biological activity of human insulin from the nanoparticles was 96.1±0.8% of the insulin content in the nanoparticle formulation. Samples were analyzed in triplicate. There was no considerable loss of biological activity observed indicating the nanoparticle formulation components and the process of making nanoparticles preserved the integrity of the three dimensional structure of human insulin. Proteins are fragile molecules with labile bonds and reactive side chains, disruption of these complex structures can lead to loss of biological activity. ELISA human insulin assay measures biologically active insulin with high degree of specificity, using a pair of mouse monoclonal antibodies. The full biological activity of a protein is dependent on preserving the integrity of its three dimensional structure. ELISA results suggest that these nanoparticles are capable of preserving biological activity of entrapped insulin in presence of hydrophobic components (stearic acid, α-tocopherol acetate) and polar organic solvent (t-butanol).

**Fig. 3:**
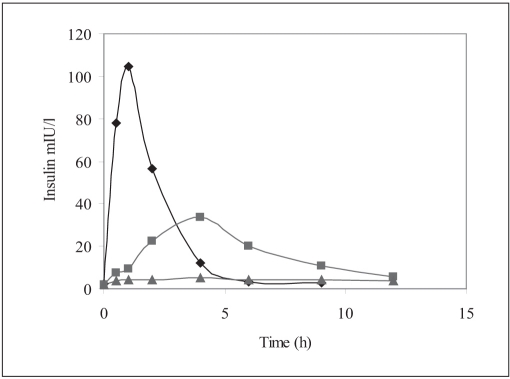
Serum human insulin versus time profile Serum human insulin levels after oral administration of 20 IU/kg Insulin nanoparticles (▪), Placebo nanoparticles equivalent to 20 IU/ kg of insulin (▲), subcutaneous injection of 2 IU/kg Human insulin solution (♦), n=6 per group.

Human insulin nanoparticles (Group-3) showed significantly high serum human insulin concentration levels over the 4 to 12 h period were observed as compared to the placebo control (Group-1). Fig. 3 shows the serum human insulin concentration profile versus time for each group.

The pharmacokinetic parameters such as C_max_, T_max_, AUC_0-t_, T_½_ were estimated for each group and the values observed are reported in Table 2. From area under human insulin concentration curves, AUC_0-12h_ for 20 IU/kg dose of nanoparticle formulation was almost similar to subcutaneous injection (SC. Inj.) of 10 IU/kg human insulin solution while the bioavailability of the latter was diminished at 6 h after injection, the former seemed to be continuing its absorption and hypoglycemic activity (fig. 3)

**TABLE 2:** PHARMACOKINETIC PARAMETERS FOR HUMAN INSULIN

Parameter	SC. Inj. Human insulin solution 2 IU/kg	Oral insulin nanoparticles, 20 IU/kg
C_max_ (mIU/l)	104.7	33.5
T_max_ (h)	1	4
AUC_0-12h_ (mIU/l/h)	189.2	178.4
K_e_	0.754	0.903
T_½_ (h)	0.919	0.767

Pharmacokinetic parameters are estimated from the each group of six male Wistar rats after subcutaneous injection of 2 IU/kg human insulin solution and oral administration of 20 IU/kg insulin nanoparticles

**Fig. 4:**
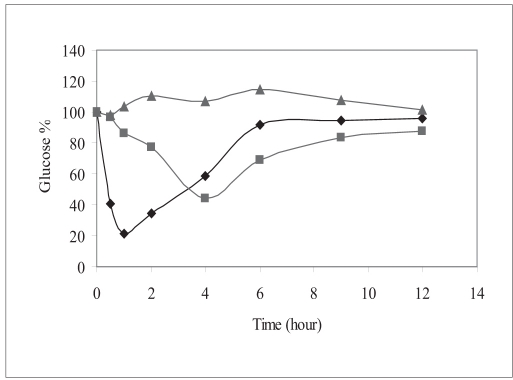
Mean plasma Glucose concentration (%) versus time curve Plasma glucose levels in percentage after oral administration of 20 IU/kg Insulin nanoparticles (▪), Placebo nanoparticles equivalent to 20 IU/kg of insulin (▲), subcutaneous injection of 2 IU/kg human insulin solution (♦), n=6 per group

**Fig. 5:**
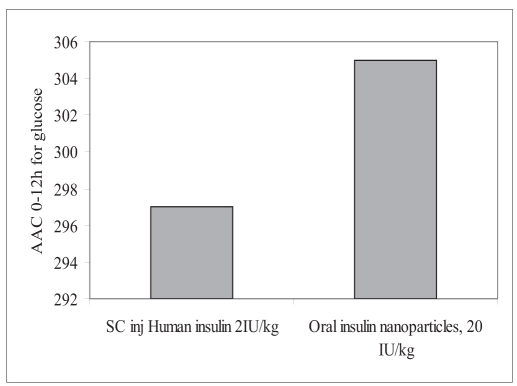
Plasma glucose AAC_0-12h_ Comparison of plasma glucose AAC_0-12h_ for intragastrically administered oral insulin nanoparticles at 20 IU/kg with subcutaneous injection of human insulin solution at 2 IU/kg.

Human insulin nanopartic1es (Group-3) significantly reduced the blood glucose levels over 4 to 12 h period compared to the placebo control (Group-1). Fig. 4 shows the blood glucose profile for each group. Hypoglycemic effect of the 20 IU/kg dose was almost similar to that with 2 IU/kg subcutaneous dose with respect to the extent of reduction in glucose concentration from base line (∼ 56% for 20 IU/kg-intragastrically administered human insulin nanoparic1es and ∼78.6% of baseline for 2 IU/kg subcutaneously injected human Insulin solution). Area above glucose concentration curves, AAC_0-12h_ calculated from these curves as depicted in fig. 5 show that the effect of 20 IU/kg dose of nanopartic1e formulation was almost similar to subcutaneous injection of 2 IU/kg insulin while the effect of the latter was diminished at 6 h after injection, the former seemed to be continuing its hypoglycemic activity.

Hydrophobic nanoparticles of insulin were found to retain the biological activity of insulin in presence of hydrophobic components (stearic acid, α tocopherol acetate and SPC). The innovative process for the preparation of nanoparticles was designed with excellent entrapment efficiency. The duration of *in vitro* insulin release was found to get extended beyond 3 h from the nanoparticles, however estimation could not be performed for extended period due to degradation of insulin in the release medium at 37°. *In vivo* study in the streptozocin induced diabetic rats showed improvement in the oral absorption of insulin. The oral dose of 20 IU/kg of nanoparticle formulation was found to be comparable with 2 IU/kg subcutaneous injection of human insulin injection in terms of AUC_0-12h_ for human insulin serum versus time profile and AAC_0-12h_ for plasma glucose % glucose reduction. C_max_ for human insulin and extent of plasma glucose reduction was lower with oral formulation as compared to subcutaneous injection of human insulin solution, but the bioavailability of the latter was diminished at 6 h after injection, the former seemed to be continuing its absorption and hypoglycemic activity.
